# Novel Pathway of Adenosine Generation in the Lungs from NAD^+^: Relevance to Allergic Airway Disease

**DOI:** 10.3390/molecules25214966

**Published:** 2020-10-27

**Authors:** Richard Graeff, Alonso Guedes, Ruth Quintana, Erin Wendt-Hornickle, Caroline Baldo, Timothy Walseth, Scott O’Grady, Mathur Kannan

**Affiliations:** 1 Department of Veterinary and Biomedical Sciences, College of Veterinary Medicine, University of Minnesota, St. Paul, MN 55108, USA; richardgraeff@gmail.com; 2 Department of Veterinary Clinical Sciences, College of Veterinary Medicine, University of Minnesota, St. Paul, MN 55108, USA; guede003@umn.edu (A.G.); rquintan@umn.edu (R.Q.); ewendtho@umn.edu (E.W.-H.); cfbaldo@umn.edu (C.B.); 3 Department of Pharmacology, University of Minnesota Medical School, University of Minnesota, St. Paul, MN 55455, USA; walse001@umn.edu; 4 Department of Animal Science, College of Food, Agricultural and Natural Resource Sciences, University of Minnesota, St. Paul, MN 55108, USA; ograd001@umn.edu

**Keywords:** CD38, asthma, airway smooth muscle, epithelial cells

## Abstract

Adenosine and uric acid (UA) play a pivotal role in lung diseases such as asthma and chronic obstructive pulmonary disease (COPD). In the present experiments, we measured adenosine synthesis from nicotinamide adenine dinucleotide (NAD^+^) in membranes prepared from wild type (WT) and CD38 knockout (CD38KO) mouse lungs, from cultured airway smooth muscle and epithelial cells, and in bronchoalveolar lavage fluid after airway challenge with epidemiologically relevant allergens. Adenosine was determined using an enzymatically coupled assay that produces ATP and is detected by luminescence. Uric acid was determined by ELISA. Exposure of cultured airway epithelial cells to *Alternaria alternata* extract caused significant nucleotide (NAD^+^ and ATP) release in the culture media. The addition of NAD^+^ to membranes prepared from WT mice resulted in faster generation of adenosine compared to membranes from CD38KO mice. Formation of adenosine from NAD^+^ affected UA and ATP concentrations, its main downstream molecules. Furthermore, NAD^+^ and adenosine concentrations in the bronchoalveolar lavage fluid decreased significantly following airway challenge with house-dust mite extract in WT but not in CD38KO mice. Thus, NAD^+^ is a significant source of adenosine and UA in the airways in mouse models of allergic airway disease, and the capacity for their generation from NAD^+^ is augmented by CD38, a major NADase with high affinity for NAD^+^. This novel non-canonical NAD^+^-adenosine-UA pathway that is triggered by allergens has not been previously described in the airways.

## 1. Introduction

Chronic obstructive lung diseases, such as asthma and chronic obstructive pulmonary disease (COPD), are the most common chronic respiratory inflammatory diseases and are among the leading causes of morbidity and mortality worldwide [1]. In the United States, these conditions are the third leading cause of death. Among Americans of all age groups, the estimated lifetime asthma prevalence is 8.4% [2] with an associated economic burden of $82 billion dollars [3]. The risk of developing COPD is largely related to modifiable behaviors (e.g., smoking), but risk factors for asthma are more heterogeneous, less understood, and more difficult to control. Symptomatic therapy (e.g., corticosteroids, bronchodilators) has significantly decreased asthma mortality, but the overall prevalence is on the rise [1], underscoring the need for a better understanding of risks and mechanisms to enable progress in therapeutic development.

The importance of nicotinamide adenine dinucleotide (NAD^+^) and its related metabolic enzymes in health and disease is becoming increasingly appreciated [4]. Our laboratory was the first to report the contribution of CD38, a type II transmembrane glycoprotein, to the regulation of intracellular calcium in airway smooth muscle (ASM) and airway hyperresponsiveness (AHR) in a series of studies employing cellular imaging, molecular and biochemical techniques in cells, tissues and animal models (e.g., [5]). CD38 was first identified as a cell-surface marker in B-cells and is now widely recognized as a multifunctional ectoenzyme that converts NAD^+^ into multiple calcium-mobilizing messengers, including cyclic ADP ribose (cADPR), ADP ribose (ADPR) and nicotinic acid adenine dinucleotide phosphate (NAADP) [5,6]. In brief, our previous studies revealed a prominent role of the CD38/cADPR signaling in heightened intracellular calcium responsiveness and contractile properties of human and mouse ASM cells exposed to inflammatory cytokines such as TNF-α, IFN-γ and IL-1β as well as the Th_2_ cytokine IL-13 [7,8,9,10,11]. Furthermore, CD38 activity in resident airway cells, rather than in inflammatory cell infiltrate, significantly contributes to airway hyperresponsiveness in mouse models of cytokine- and allergen-induced inflammatory airway disease [12,13,14].

Adenosine and uric acid have a central role in mediating the pathophysiology of chronic obstructive lung diseases [15,16,17,18,19,20,21,22,23,24,25,26,27,28,29,30,31]. The role of adenosine in asthma has been investigated in the context of the subtype(s) of adenosine receptors involved in the process and in the design of selective antagonists of adenosine receptors to mitigate its effects in the airways [24,29,30,32,33,34]. Prior investigations have focused on the role of UA in innate immunity and its role in the asthmatic phenotype in mouse models as well as in human asthma [15,16,17,19]. While extracellular ATP is the main source of adenosine, which can be further metabolized to uric acid in a multistep pathway, CD38-generated ADPR from NAD^+^ was recently shown to form a non-canonical adenosinergic pathway in human Jurkat cell line [35]. Thus, in the present study, we aimed to determine whether CD38 is involved in a non-canonical pathway of adenosine synthesis from NAD^+^ in the lungs. To address this hypothesis, we used primary human airway smooth muscle (HASM) cells in short-term culture to maintain a differentiated smooth muscle contractile phenotype, human bronchial epithelial cells (16HBE) in immersion culture and whole mouse lungs in models of allergic airway diseases.

## 2. Results

### 2.1. Adenosine Assay

**Figure 1:**
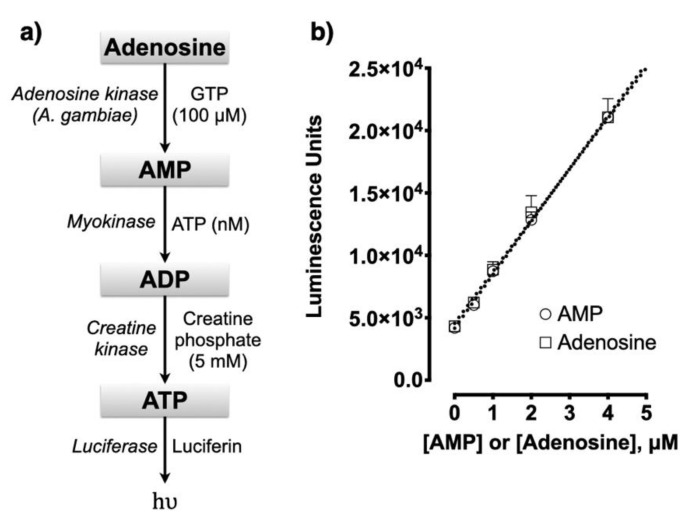
(**a**) Adenosine assay. The complete assay includes adenosine kinase, myokinase, and creatine kinase for the conversion of adenosine to ATP. Adenosine concentration is determined from the difference in luminescence in the absence and presence of adenosine kinase. Enzymes are listed on the left of arrows and substrates listed on the right. (**b**) Standard curves for AMP and adenosine.

As described in the methods section, adenosine was measured by sequential conversion of adenosine to ATP, as in the multistep pathway known as de novo synthetic pathway. Each step was sequentially catalyzed by the specific enzymes adenosine kinase, myokinase, and creatine kinase and the final ATP product was determined using luminescence assay (Figure 1a). The adenosine kinase, containing a HIS tag, was expressed in *E. coli* and purified with a nickel column. The myokinase and creatine kinase were from commercial sources. As shown in Figure 1b, the standard curves for adenosine and AMP were essentially the same after 3 h of incubation. The lower limit of detection ranged between 10 and 100 nM. The incubation conditions for AMP measurement include myokinase and creatine kinase. The concentration of AMP is calculated from the difference in luminescence between the absence and presence of myokinase. The concentration of adenosine is calculated from the difference in luminescence between the absence and presence of adenosine kinase. Adenosine kinase has no effect on the AMP measurement. Although GTP is added to the reagent to support the adenosine kinase reaction, no added ATP is included for the myokinase reaction. It is assumed that there is sufficient ATP (nM) in the reagent for this purpose.

### 2.2. CD38 Activity in ASM Cells Contributes to a Non-Canonical Pathway of Adenosine Synthesis

**Figure 2:**
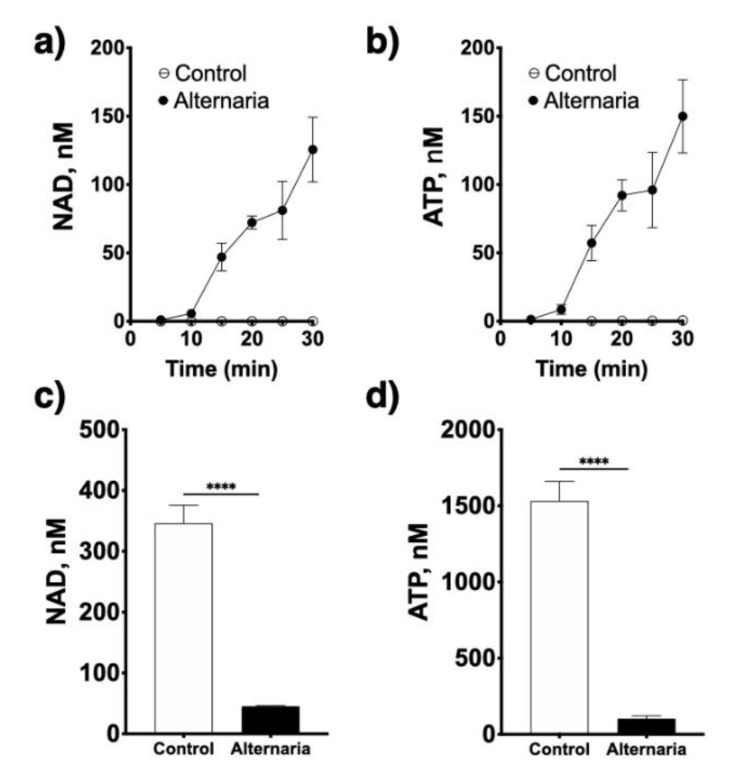
Allergen exposure induces rapid nucleotide release from airway epithelial cells. Cultured human bronchial epithelial cells rapidly release NAD^+^ (**a**) and ATP (**b**) into the medium upon exposure to an extract of *Alternaria alternata* (50 μg/mL), and resulting in rapid decrease in intracellular NAD^+^ (**c**) and ATP (**d**) content. **** *p* < 0.0001, two-tailed unpaired *t* tests; *n* = 4 replicates/treatment.

**Figure 3:**
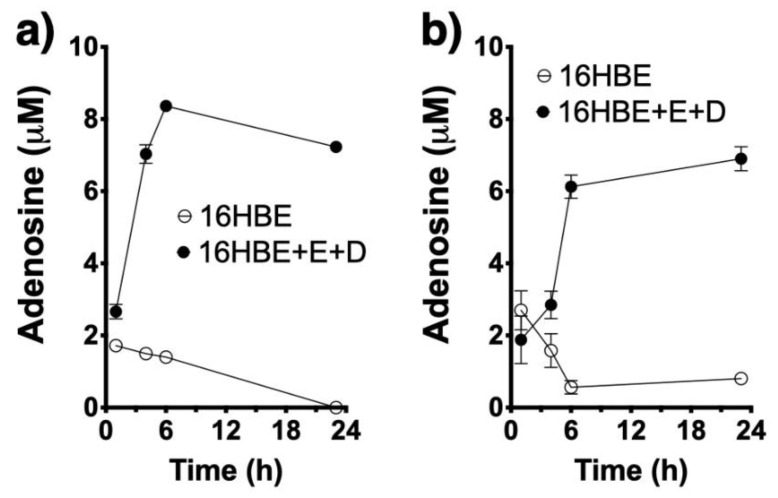
Airway epithelial cells synthesize adenosine from NAD^+^ and ATP. Adenosine synthesis from 100 μM NAD^+^ (**a**) or ATP (**b**) in the presence or absence of the adenosine deaminase (EHNA, E) and adenosine reuptake (dipyridamole, D) inhibitors (*n* = 4 replicates) in airway epithelial (16HBE). Note that addition of the two inhibitors is required to enable detection of adenosine accumulation.

CD38 is a multifunctional enzyme that catalyzes the synthesis of cADPR from NAD^+^ (cyclase activity) and of ADPR either from cADPR (hydrolase activity) or directly from NAD^+^ (NADase activity). cADPR induces calcium release from ryanodine-sensitive stores in ASM cells (14). Our previous work delineated the role for the cyclase activity of CD38 whereby its product cADPR mediates calcium hyperresponsiveness of human ASM treated with asthma-relevant cytokines [7,8,9,10], and established the contribution of CD38 to airway hyperreactivity to contractile agonists in cytokine and mouse models of allergic airway disease [12,13,14]. However, its other enzymatic activities, both of which result in the production of ADPR either directly or indirectly from NAD^+^, have not been previously explored in the context of allergic airway diseases. Thus, in the present study, it was of interest to determine whether NAD^+^ serves as a precursor to form adenosine in the lungs, another potential trigger of ASM contraction in asthma. First, we determined if allergen exposure could trigger nucleotide release from human airway epithelial cells. This is important because NAD+ and ATP are primarily stored intracellularly but the nucleotide-metabolizing enzymes required for adenosine synthesis (i.e., CD38, CD203a and CD73) are all ectoenzymes, thus requiring enough substrate to be available extracellularly. Cultured 16HBE cells treated with an extract of the fungal allergen *Alternaria alternata* rapidly released NAD^+^ as well as ATP in the culture media (Figure 2), and these cells were capable of converting both NAD^+^ and ATP to adenosine (Figure 3). However, CD38 enzymatic activity was not present in the 16HBE cells (not shown), suggesting that other NADases (e.g., CD203a, PARP-1) are active in epithelial cells.

**Figure 4:**
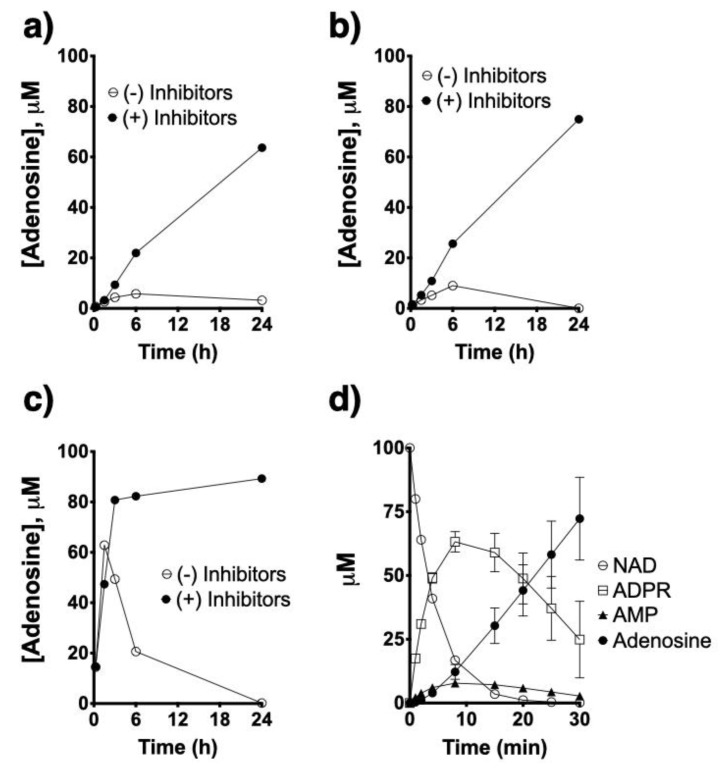
Comparison of ectoenzyme activities in human airway smooth muscle. Cultured primary human airway smooth muscle cells from asthmatic subjects (*n* = 4 replicates) were treated with 100 μM NAD^+^ (**a**), ADPR (**b**) and AMP (**c**) in the presence or absence of adenosine deaminase and re-uptake inhibitors and adenosine formation was determined. Note no/minimal adenosine accumulation in the absence of the inhibitors. Also note the rate of adenosine production from NAD^+^ and ADPR is similar, while that from AMP is markedly faster. The kinetics of synthesis of ADPR, AMP and adenosine from NAD^+^ is shown in (**d**).

Using HASM cells obtained from asthmatic subjects, in which CD38 activity is readily detectable under control conditions and especially following exposure to TNF-α [7,8,9], we compared activities of the different ectoenzymes present in HASM cells that are involved in the nucleotide metabolic pathway. HASM cells were able to synthesize adenosine from added NAD^+^, ADPR or AMP (Figure 4). Use of inhibitors of adenosine deaminase (EHNA) and of adenosine reuptake (dipyridamole) was required to enable detecting adenosine accumulation. Significantly, the HASM produced adenosine from NAD^+^ (Figure 4a) or ADPR (Figure 4b) at a similar rate, while production of adenosine from AMP (Figure 4c) occurred at a markedly faster rate and resulted in greater total adenosine production during the 24 h incubation time. As shown in Figure 4d, NAD^+^ was consumed very rapidly with a corresponding rise in ADPR concentrations that tapered off and then decreased as adenosine was rising, while AMP accumulations were relatively small. Collectively, the above results confirmed that allergen exposure triggered nucleotide release from airway epithelial cells, and that ectoenzymes present in both airway epithelial and smooth muscle cells have the ability to metabolize such nucleotides to adenosine. In addition, the results suggest that the ectoenzyme pyrophosphatase CD203a constitute a rate limiting step in the multistep synthetic pathway from NAD^+^ to adenosine.

**Figure 5:**
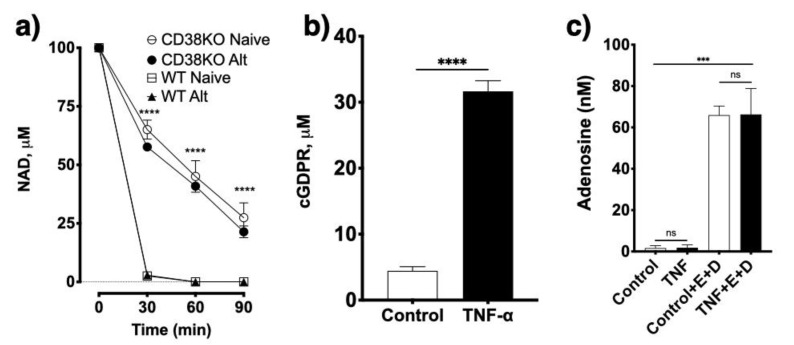
Effect of loss or gain of CD38 function on NAD^+^ metabolism and adenosine synthesis. (**a**) Consumption of NAD^+^ (100 μM) was measured using lung tissue homogenates from wild type (WT) and CD38 knockout (CD38KO) mice (*n* = 4/group) under naive conditions (Naive) or following intranasal challenge with an extract of the fungal allergen *Alternaria alternata* (Alt; 50 μg in 50 μL saline). Note that by 30 min all the NAD^+^ had been consumed in WT mice whereas only approximately 30–40 μM had been consumed in the CD38KO lung tissues. **** *p* < 0.0001 between WT and CD38KO mice, irrespective of allergen treatment. Mixed-effect model. (**b**) Human airway smooth muscle cells obtained from asthmatic subjects were incubated with TNF-α (10 ng/mL) for 24 h to up-regulate CD38 enzymatic activity. CD38 activity was determined fluorometrically using nicotinamide guanine dinucleotide (NGD) as substrate (100 μM) and production of cGDPR determined at 300/410 excitation/emission wavelengths with a microplate reader. **** *p* < 0.0001 compared to vehicle control treatment; two-tailed unpaired *t*-test. (**c**) TNF-α (10 ng/mL) or inhibitors of adenosine deaminase (EHNA, E) and of adenosine reuptake (dipyridamole, D) were added 24 h before the adenosine was measured, as described in the methods section. Note that upregulation of CD38 activity did not result in further adenosine production compared to control treatment and the need of including the inhibitors to enable detection of adenosine accumulation. ns = not significant, *** *p* < 0.001 compared to respective vehicle control treatment; ANOVA and Bonferroni post-hoc. Cell data in panels b and c are from *n* = 4 independent experiments in triplicate. Data are Mean ± SEM.

**Figure 6:**
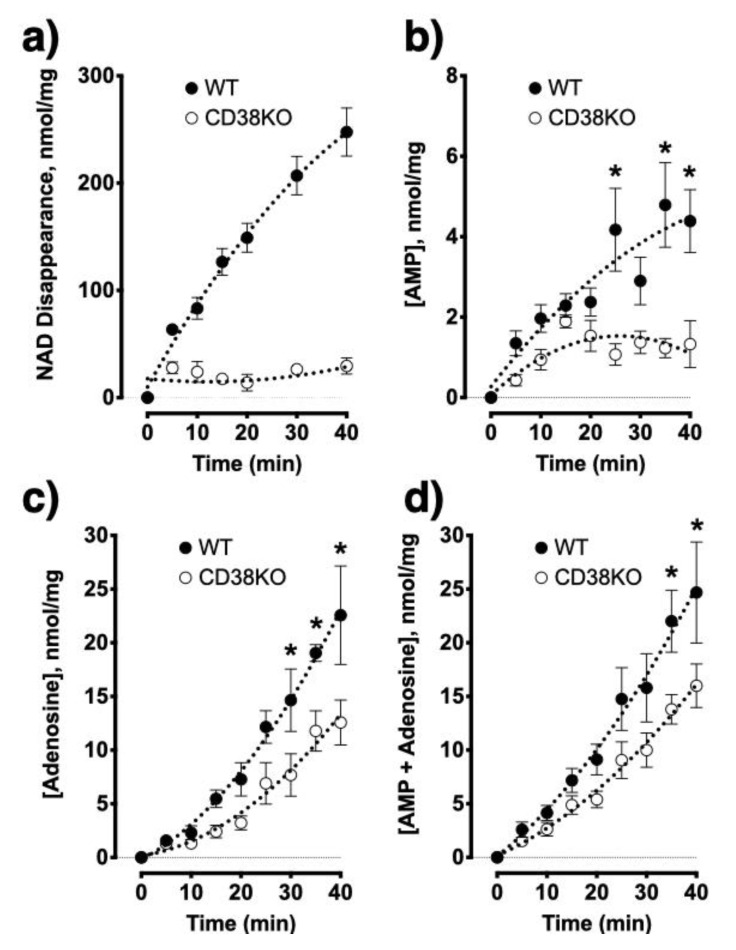
Genetic loss of CD38 impairs AMP/adenosine synthesis from NAD^+^ in the lungs. Lung homogenates (*n* = 4/group) from wild-type (WT) mice rapidly degrade NAD^+^ whereas minimal NAD^+^ degradation occurs in tissues from CD38KO (KO) mice (**a**). Tissues from WT mice synthesize both AMP (**b**) and adenosine (**c**) in greater amounts than tissues from KO mice. (**d**) The sum of AMP and adenosine formation is markedly greater in lungs of WT mice. * *p* < 0.05; One-way repeated measures ANOVA and Bonferroni post-hoc.

**Figure 7:**
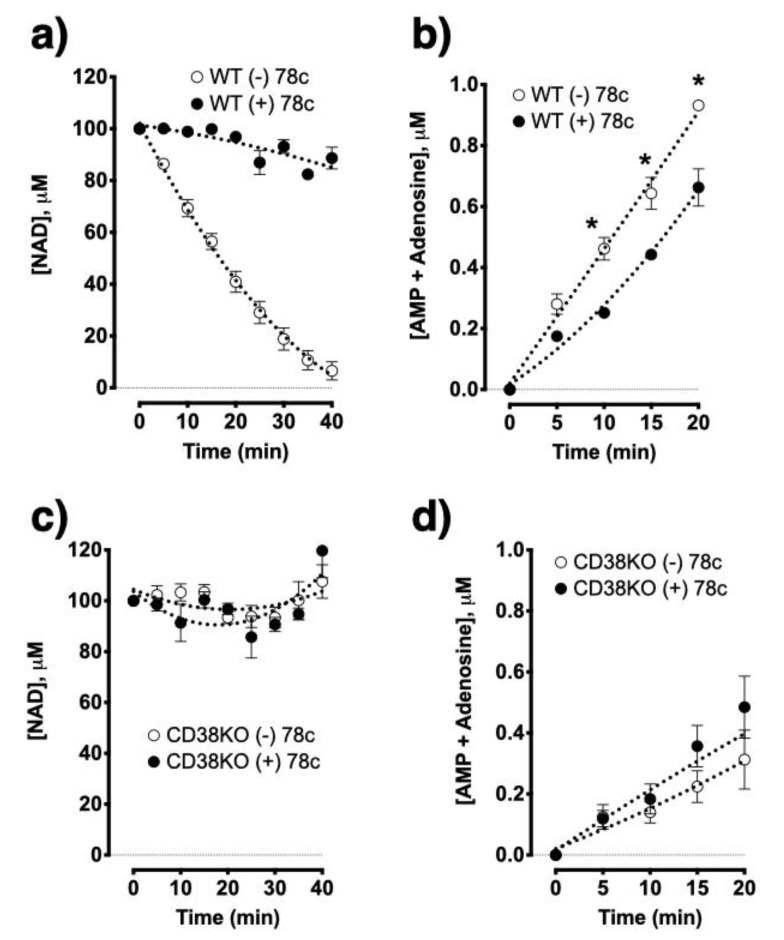
Pharmacologic inhibition of CD38 blocked NADase activity and impaired nucleotide synthesis in wild type but not in CD38KO mouse lungs. Metabolism of NAD^+^ was blocked by the CD38 inhibitor compound 78c (200 nM) in the lungs of wild type (WT) mice (**a**), resulting in lower nucleotide synthesis from NAD^+^ (100 μM) (**b**), whereas it did not affect NAD^+^ metabolism (**c**) and nucleotide synthesis (**d**) in the lungs of CD38KO mice. * *p* < 0.05; One-way repeated measures ANOVA and Bonferroni post-hoc; *n* = 4 mice/group.

**Figure 8:**
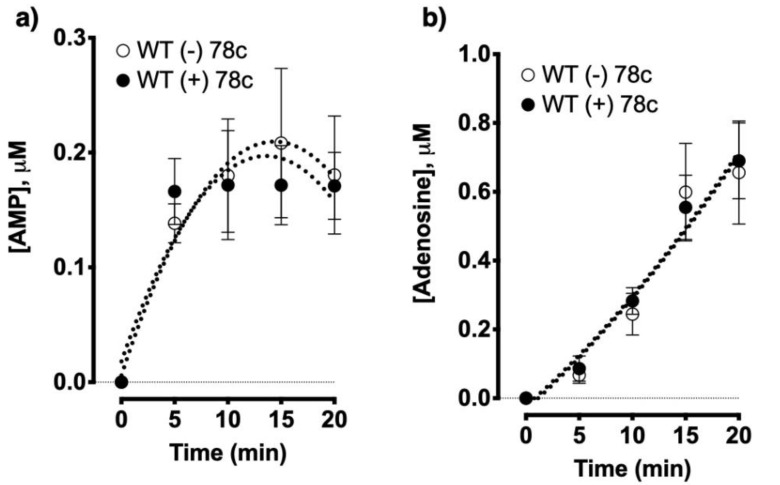
Pharmacologic inhibition of CD38 does not affect nucleotide synthesis from ADP ribose. Lung homogenates from wild type (WT) mice (*n* = 4/treatment) were treated with the CD38 inhibitor compound 78c (200 nM) or vehicle control and the synthesis of AMP (**a**) and adenosine (**b**) were monitored.

To delineate whether allergen exposure affects CD38 and/or other NADase activities in the lungs, we intranasally challenged WT and CD38KO mice with an extract of the fungal allergen *Alternaria alternata* as previously reported by our laboratory [12]. The total lung NADase activity was markedly impaired in CD38KO mice as compared to WT controls, but the activity was not altered in either mice following allergen exposure (Figure 5). Thus, NAD^+^ consumption in the lungs is profoundly affected by CD38 activity with no further changes triggered by allergen exposure, suggesting that CD38 is the principal NADase in the lungs. Using HASM cells, we upregulated CD38 expression by treating the cells with TNF-α and measured adenosine production from NAD^+^. In spite of a marked increase in CD38 enzyme activity induced by TNF-α treatment, there were no associated increases in adenosine production indicating that basal CD38 activity is sufficient for maximal conversion of NAD^+^ to adenosine, again consistent with the notion that activity of another enzyme (CD203a) constitute a rate-limiting step. Next, to examine the impact of impaired CD38 NADase activity on nucleotide/nucleoside synthesis, we simultaneously monitored NAD^+^ disappearance along with AMP and adenosine formation using lung membranes prepared from WT and CD38KO mice. As predicted, loss of the NADase activity of CD38 significantly impaired NAD^+^ consumption as well as the synthesis of AMP and adenosine (Figure 6). These results were corroborated by experiments using compound 78c, a pharmacologic inhibitor of CD38 enzymatic activity [36,37], which significantly decreased NAD^+^ consumption and AMP and adenosine production by WT but not CD38KO lung membrane homogenates (Figure 7). Furthermore, pharmacologic inhibition of CD38 did not block synthesis of adenosine and AMP from added ADPR, consistent with the role of CD38 in this non-canonical pathway of adenosine synthesis (Figure 8). We determined NADase activity in bronchoalveolar inflammatory cell infiltrate obtained from allergen-challenged mice using a cycling assay [38] but found no detectable NADase activity (not shown). These data suggest that lung NAD^+^ metabolism during allergen challenge is carried out primarily by lung parenchymal cells and not by the recruited bronchoalveolar inflammatory cells.

### 2.3. NAD^+^ Contributes to Adenosine Synthesis and Affect both UA and ATP Concentrations in the Lungs

**Figure 9:**
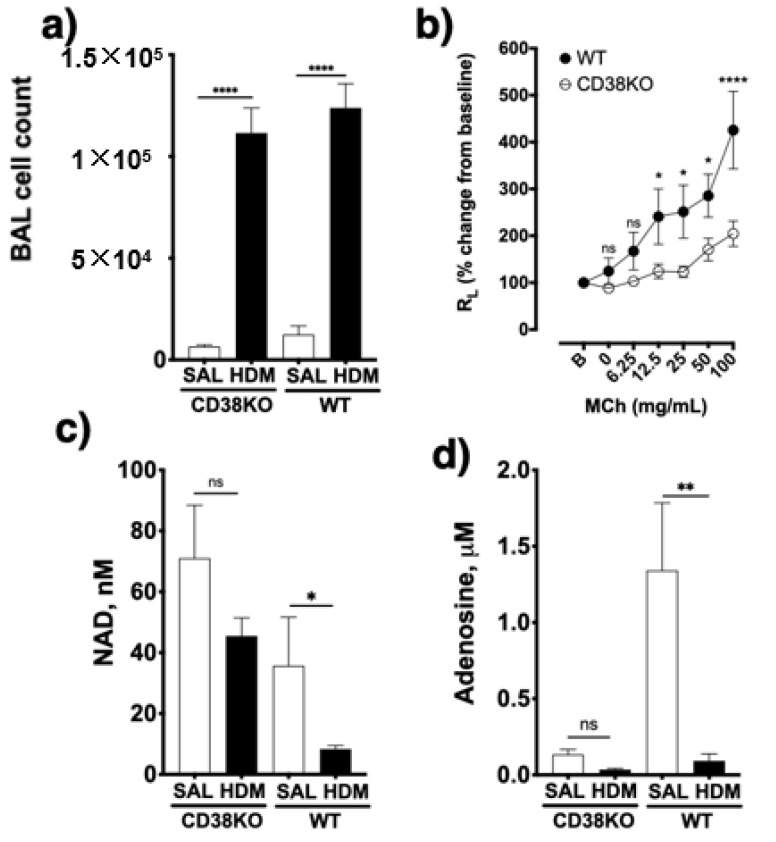
CD38 activity enhances allergen-induced airway hyperresponsiveness and affect bronchoalveolar levels of NAD^+^ and adenosine. (**a**) Wild-type (WT) and CD38KO mice (*n* = 6–12/group) were intranasally challenged with house-dust mite (HDM) extract for 2 weeks. Allergen challenge significantly increased the inflammatory cell numbers in the bronchoalveolar lavage (BAL) fluid compared to saline (SAL)-challenged WT and CD38KO mice. (**b**) Airway responsiveness to inhaled methacholine was significantly greater following allergen challenge in WT than in CD38KO mice. (**c**) Allergen challenge significantly decreased NAD^+^ concentrations in the BAL fluid in WT but not in CD38KO mice. (**d**) Adenosine concentrations decreased significantly in BAL fluid in WT but not in CD38KO mice. ns = not significant, * *p* < 0.05, ** *p* < 0.01, **** *p* < 0.0001; One-way ANOVA (**a**), (**c**), (**d**) or mixed-effect model (**b**) with Bonferroni post-hoc.

**Figure 10:**
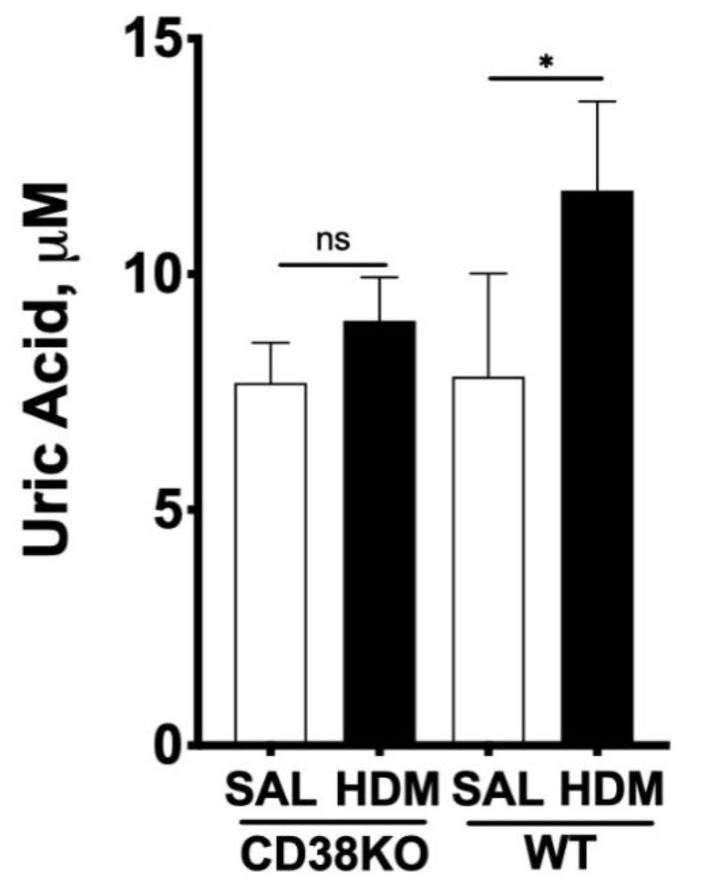
CD38 activity is associated with elevated uric acid concentration in bronchoalveolar fluid during allergic airway inflammation. Wild-type (WT) and CD38 knockout (CD38KO) mice (*n* = 6–12/group) were intranasally challenged with house-dust mite (HDM) extract for 2 weeks. Allergen challenge was associated with significantly greater increase in uric acid levels in WT but not in CD38KO mice. * *p* < 0.05; One-way ANOVA with Bonferroni post-test, ns = not significant.

**Figure 11:**
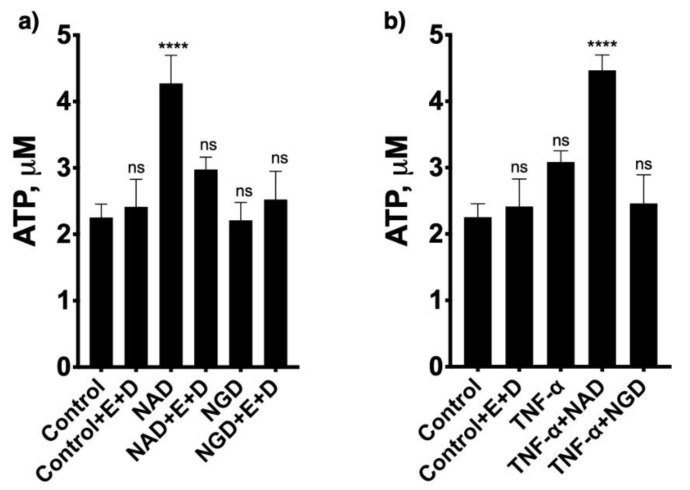
Effect of NAD^+^ on intracellular ATP concentration in human airway smooth muscle cells. Cells obtained from asthmatic subjects were grown in standard DMEM media containing 10% fetal calf serum and antibiotics. Culture conditions were changed to an arresting medium for 96 h, which contained 5.7 μg/mL insulin, 5 μg/mL transferrin, antibiotics, and no serum. (**a**) Cells were incubated for 24 h with 100 μM NAD^+^, NGD or their vehicle control with and without EHNA (E) and dipyridamole (D) and intracellular ATP concentrations were determined using a chemiluminescence assay. (**b**) 100 μM NAD^+^ or NGD was added at 24 h of treatment with TNF-α and incubated with cells in culture with and without EHNA (E) and dipyridamole (D) for an additional 24 h, when intracellular ATP concentrations were determined. *n* = 4 independent experiments. ns = not significant, **** *p* < 0.0001 compared to vehicle control treatment; ANOVA and Bonferroni post-hoc. Mean ± SEM of *n* = 4 independent experiments).

**Figure 12:**
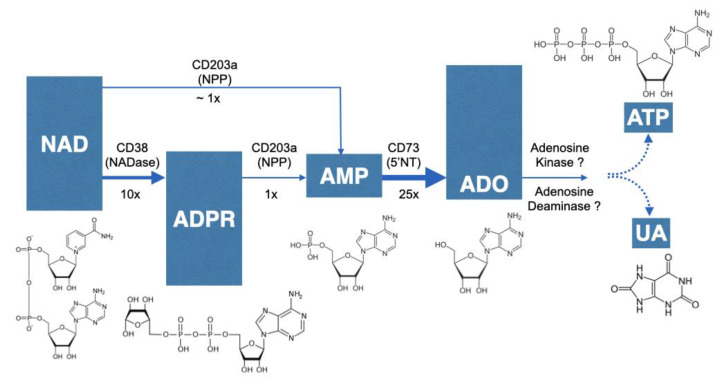
Metabolism of NAD^+^ with membranes from lung or after incubation of ASM cells. NAD^+^ is converted to ADPR by CD38. In the absence of CD38, NAD^+^ is metabolized to AMP by a pyrophosphatase activity such as CD203a. ADPR is also converted to AMP by CD203a. The conversion of NAD^+^ or ADPR to AMP is much slower than that of NAD^+^ to ADPR by CD38. Once AMP is formed, it is rapidly converted to adenosine (ADO) by CD73. Based on results of cells in culture and incubations of membranes with various substrates, the rate limiting step of these reactions is at the CD203a step. Formed adenosine can be recycled intracellularly to the ATP pool or be metabolized to uric acid (UA). Chemical structures are shown.

To confirm the role of CD38 to NAD^+^ consumption and adenosine synthesis during allergic airway inflammation, mice were intranasally challenged with house dust mite, another epidemiologically relevant aeroallergen, as described in the methods section. As shown in Figure 9, allergen challenge caused marked increases in bronchoalveolar inflammation in both WT and CD38KO mice, but airway hyperresponsiveness to inhaled methacholine was significantly greater in the WT than in CD38KO mice, consistent with previous studies from our laboratory [12,13,14]. The BAL concentrations of NAD^+^ were higher in CD38KO than in WT mice following saline challenge. Although allergen challenge caused the NAD^+^ concentrations to decrease in both mice, this was significant in the WT but not in CD38KO mice. These results are coherent with the loss of a major NADase (CD38) in lung tissues. The BAL adenosine concentrations in saline-challenged mice were higher in WT compared to CD38KO, also in line with the NAD^+^ findings. However, the higher NAD^+^ consumption did not directly translate to increased adenosine levels in the BAL of either mice but, instead, BAL adenosine concentrations decreased in both mice, significantly in the WT but not in CD38KO mice. A major metabolite of adenosine being uric acid (UA), we determined its concentrations in the BAL fluid. As shown in Figure 10, the UA concentrations in BAL fluid in saline-challenged mice was similar between WT and CD38KO whereas allergen challenge significantly increased BAL fluid UA concentrations in WT but not in the CD38KO. Collectively, the data suggest that CD38 activity is associated with faster NAD^+^ metabolism to adenosine and UA during allergic airway inflammation. Since formation of UA is not the only fate of adenosine, we used HASM cells to determine whether addition of NAD^+^ (100 μM) influenced the levels of intracellular ATP. In the absence of adenosine deaminase inhibition with EHNA and of adenosine re-uptake with dipyridamole, but not in their presence, addition of NAD^+^ caused a 79 ± 29% increase in the intracellular ATP pool (Figure 11), suggesting that the NAD^+^-generated adenosine can also be converted to ATP intracellularly. A similar increase in ATP was obtained in HASM cells treated with TNF-α, suggesting that upregulation of CD38 activity is not required for maximal effect and that basal levels are sufficient for NAD^+^ conversion to ATP, in line with the previous results (Figure 5). No change in intracellular ATP was obtained with addition of NGD, which is efficiently converted to cGDPR by CD38, confirming that the adenine (or adenosine) from NAD^+^ was recovered in the cellular ATP pool. Thus, these results suggest that at least some of the adenosine produced from NAD^+^ is recycled intracellularly to ATP. Collectively, our findings indicate that CD38-mediated NAD^+^ metabolism is a significant contributor to adenosine synthesis, which can influence UA concentrations in the airways as well as intracellular ATP levels in airway smooth muscle. A model depicting the metabolism of NAD^+^ with membranes from lungs or after incubation of ASM cells is shown in Figure 12. 

## 3. Discussion

The first evidence of a non-canonical adenosinergic pathway involving CD38 NADase activity was described in human Jurkat T cell line [35]. In the present study, we generated evidence for this non-canonical pathway of adenosine generation in the lungs, demonstrating for the first time its presence in primary mammalian cells and tissues. We found that 16HBE cells, which are widely used as surrogates for native human airway epithelium, exposed to *Alternaria alternata* extract rapidly released large amounts of NAD^+^ and ATP (intracellular levels decreased by 87% and 93%, respectively) indicating that exposure to aeroallergens would increase substrate availability for local adenosine synthesis. Indeed, addition of equimolar concentrations of NAD^+^ and ATP to 16HBE cells resulted in comparable amounts of adenosine formation. Further, we identified significantly lower NAD^+^ conversion to AMP and adenosine in lung tissues from CD38KO mice compared to lungs of WT controls. These findings were corroborated with a pharmacologic inhibitor of CD38. As anticipated, the CD38 inhibitor did not affect the conversion of added ADPR, a CD38 metabolite of NAD^+^, to AMP and adenosine, further confirming the role of CD38 in the NAD^+^ conversion pathway to adenosine. In these studies, we used a single concentration of NAD^+^ (100 μM), which is expected to optimally drive the NADase activity of CD38 but possibly not that of CD203a given the widely different K_m_ for these enzymes (48 vs. 330 μM, respectively) [39,40]. Under these conditions, the amount of product resulting from CD38 NADase activity was greater than that achieved by all other NADases (1000 nM vs. 600 nM). In the extracellular milieu, where NAD^+^ concentrations (nM range) are expected to be lower than used in our experimental condition (μM), CD38 is likely to be the dominant NAD^+^-consuming enzyme. Interestingly, increasing CD38 enzyme activity with TNF-α in HASM or during allergic airway inflammation induced by *Alternaria alternata* in mice did not augment NAD^+^ consumption over basal conditions, suggesting that basal NADase activity, primarily by constitutively expressed CD38, is sufficient for maximal NAD^+^ metabolism. Thus, CD38 inhibition is expected to greatly affect NAD^+^ consumption in the lungs under normal and allergic conditions. However, in terms of its effects on adenosine, the functional impact may be greater under pathologic conditions given the overexpression of adenosine and its receptors in patients with chronic obstructive pulmonary diseases [20,25] and that the bronchoconstrictive actions of adenosine occurs in asthmatics but not in non-asthmatic subjects [32]. Collectively, our findings provide unambiguous evidence for a non-canonical NAD^+^-dependent adenosinergic pathway in the lungs, implicating CD38 as a major contributor to this process with potential therapeutic value in chronic obstructive pulmonary diseases.

A comparison of ectoenzyme activities in HASM was made directly with cells in culture. We determined the relative activities of CD38, CD203a and CD73 by incubating the cells with equimolar concentrations of their respective substrates (NAD^+^, ADPR, and AMP) while monitoring adenosine production in the culture media. By comparing their apparent kinetics, it can be inferred that CD203a activity is a rate-limiting step in the multistep pathway of adenosine synthesis in HASM cells. Additionally, the ASM is likely the cell type with the greatest capacity to convert NAD^+^ to adenosine in the lungs because it appears to be the primary if not the only cell type with detectable CD38 activity [8,9,10,41,42,43,44,45,46,47,48]. CD38 is expressed in very low numbers of BAL cells (>10%) from healthy human subjects [49] and we were unable to detect its activity in the mouse bronchoalveolar inflammatory cell infiltrate. We were also not able to detect CD38 activity in 16HBE cells in culture, although this does not fully exclude the possibility that CD38 is expressed in primary normal human bronchoepithelial cells [49]. The role of CD38-mediated events in the pulmonary epithelium is mostly unknown, but our results suggest that the presence of CD38 in ASM or the airway epithelium could influence mucous hypersecretion during allergic airway disease via autocrine and paracrine adenosinergic signaling mechanisms, similar to that previously described for NAD^+^ and cADPR in other model systems [50,51,52]. Indeed, separate studies using the ovalbumin model of allergic airway disease have demonstrated that mucus production is dependent on the presence of CD38 in structural lung cells and not on cells of immune lineage [53], and adenosine receptor activation causes significant mucin secretion [54]. Adenosine leads to mucous hypersecretion and is involved in other prominent features of allergic airway diseases such as hyperresponsiveness [22,29,32] and remodeling [27,30].

Our results showed that NAD^+^ contribution to lung adenosine synthesis can also impact the formation of downstream molecules such as UA and of intracellular ATP. The allergen-induced accumulation of UA in the BAL fluid of WT mice of the current study was comparable to the previously reported increased UA release in the airways of asthmatics and mice following allergen exposure [19]. However, our findings revealed an additional contribution of CD38 NADase activity to the accumulation of UA in the airways. Uric acid is involved in the development of type-2 immune response to aeroallergens [16,17,19]. A clear link between UA accumulation in the airways and the asthmatic phenotype (i.e., Th_2_-type cellular and cytokine profile and airway hyperresponsiveness) was elegantly demonstrated in a well-designed study using HDM mouse models and human asthmatics [19]. The most striking finding of that study was the marked UA release upon primary exposure to HDM allergen (i.e., without prior sensitization) as well as upon HDM challenge in sensitized asthmatics and mice [19]. In the current study, the addition of NAD^+^ to HASM cells also resulted in a significant increase in cellular ATP in the absence of dipyridamole and EHNA but not in their presence. Extensive literature shows that adenosine, UA [15,16,17,19,24,29,30,32,33,34] and ATP [55,56,57,58] are implicated in airway inflammation, remodeling and hyperresponsiveness, which are hallmark features of allergic airway diseases [5,59]. Additional studies are necessary to understand the multiple putative pathways, including their regulation and potential topological paradoxes, involved in the generation of UA and ATP from NAD^+^-derived adenosine in the context of chronic obstructive lung diseases. Our previous studies have uncovered a prominent role of CD38 in heightened calcium responsiveness in HASM obtained from asthmatic and non-asthmatic patients, in airway hyperresponsiveness in mouse models of cytokine- and allergen-induced airway disease [7,8,9,10,12,13,14], and described many of the regulatory mechanisms controlling CD38 expression and enzyme activity in HASM [41,42,43,44,45,46,47,48]. The current findings expand this understanding and introduce new opportunities for further research and novel therapeutic developments for allergic airway diseases.

## 4. Materials and Methods

Allergen extracts used in the proposed studies were of the highest quality and purity as supplied by the vendor, and from the same batch to avoid batch-to-batch constituent variability. Human bronchial epithelial cell line was from Millipore Sigma (16HBE14o- Human Bronchial Epithelial Cell Line; Cat# SCC150). This immortalized cell line was produced from bronchial epithelial cells extracted from a one-year-old male heart-lung patient. The immortalized cells retain the characteristics of normal differentiated bronchial epithelial cells and the unique identity of the cell line in each batch is confirmed by genotyping. Male and female (equal numbers per experiment) wild-type C57BL/6 and CD38 knockout (KO) mice (8–12-week old) used in the studies were produced by in house breeding. Mice were maintained in specific pathogen-free facilities in a temperature- and humidity-controlled environment in a 12:12h light:dark cycle, were fed normal rodent chow and received water *ad libitum.* The experimental protocols involving animals were reviewed and approved by the University of Minnesota Institutional Animal Care and Use Committee (Protocol ID: 1508-32867A).

### 4.1. Chemicals and Reagents

Adenosine kinase plasmid was obtained from Addgene. The ATPlite kit was from Perkin Elmer. (Waltham, MA, USA) pDEST-14, TOP10 and BL21-A1 were from ThermoFisher Scientific (Waltham, MA, USA). Nickel beads were purchased from Genesee Scientific (El Cajon, CA, USA). Bradford reagent was from BioRad (Hercules, CA, USA). EHNA was from Santa Cruz Biotechnology (Dallas, TX, USA). Unless listed otherwise, chemicals were purchased from Sigma-Aldrich. *Alternaria alternata* and house dust mite (*Dermatophagoides farinae*) extracts were obtained from Greer Laboratories Lenoir, North Carolina.

### 4.2. Cell Cultures

Human airway smooth muscle (HASM) cells: Isolation, culture and maintenance of HASM cells were carried out as described in our previous publications [41,42,43,44,45,46,47,48]. Briefly, HASM cells obtained from unidentified fatal asthmatic donors were maintained in culture no longer than the 5th passage to ensure maintenance of smooth muscle phenotype. The HASM cells were grown in DMEM supplemented with 10% fetal bovine serum (FBS), 100 U/mL penicillin, 0.1 mg/mL streptomycin, and 0.25 g/mL amphotericin B in a 37 °C humidified atmosphere containing 5% CO_2_. When cells reached approximately 80% confluence, they were growth-arrested in medium containing transferrin and insulin in the absence of serum. Cells (2.5 × 10^5^ cells/well) grown in 6-well plates were exposed to TNF-α (10 ng/mL) for 24 h following growth-arrest depending on the experiment to induce maximum expression of CD38. At different time points following the addition of 100 μM (final concentration) NAD^+^, ADPR, ATP or AMP, adenosine production was determined in culture medium and cell extracts. Media samples were obtained by removing 100 μL and adding 10 μL of 1 M HCl. At the end of the incubation period, acid extracts of cells were obtained by removing the medium and replacing it with 1 mL of 100 mM HCl. Prior to assay of adenosine or nucleotides, the pH of the samples was adjusted to 7.5 with 1 M Tris base. For the measurement of adenosine levels, cells were incubated with an inhibitor of adenosine deaminase (EHNA; 25 μM) and an adenosine uptake blocker (Dipyridamole; 2.5 μM).

Human bronchial epithelial (16HBE) cells: Cells were grown in 12-well plates in MEM with 10% FBS, 100 U/mL penicillin, 0.1 mg/mL streptomycin in a 37 °C humidified atmosphere containing 5% CO_2_. When the cells reached confluence, the medium was replaced with 2 mL of HBSS containing 11 mM glucose, 10 mM HEPES, 2.5 mM CaCl_2_, 1.2 mM MgCl_2_, pH 7.4 and the cells were maintained at room temperature for 30 min. A solution containing *Alternaria alternata* (2 mg/mL) was prepared in HBSS and 100 μL was added at 30 min. Control cells were treated with the same volume of HBSS. Samples of 100 μL of HBSS media were removed at various times and added to 10 μL of 1 M HCl and maintained at 4 °C until they were assayed for NAD and ATP. NAD was measured by a fluorescence assay and ATP was measured with the ATPlite kit (Perkin Elmer), as described below.

### 4.3. Mouse Models of Allergic Airway Disease

Wild type and CD38KO mice were briefly anesthetized with isoflurane and intranasally challenged with saline (35 μL) or house-dust mite (HDM; 25 μg in 35 μL of saline) daily for 5 days, followed by 2 days rest, and repeated for 4 more days as previously described by others [18]. For sensitization with the *Alternaria alternata* extract (50 μg in 50 μL of saline), mice were exposed intranasally to the allergen extract on days 1, 4, and 7 as described in our previous studies [12]. Twenty-four hours after the last intranasal challenge with HDM allergen, mice were used for the assessment and quantification of inflammatory cell numbers, NAD^+^ and adenosine levels in bronchoalveolar lavage (BAL) fluid. To determine NAD^+^, adenosine and uric acid levels in BAL cells, mice were anesthetized with tribromoethanol, the trachea was cannulated via tracheostomy and BAL was performed with 1 mL aliquot of ice-cold 0.9% NaCl. Total cell numbers were determined, and the cells were subsequently separated by centrifugation (4 °C, 1200× *g* for 5 min) and used for determining CD38 enzyme activity. The supernatant was used to determine NAD^+^, adenosine and uric acid concentrations. The lungs were collected and used for measurement of adenosine formation as well as for measurement of CD38 enzyme activity.

### 4.4. Membrane Preparation from Mouse Lungs

For preparation of membranes, a lung was removed and suspended in 2 mL of ice-cold buffer A (50 mM Tris/50 mM NaCl, pH 7.5) and homogenized with a tissue homogenizer. Membranes were prepared by centrifugation at 10,000 rpm for 30 min and the supernatants were removed. The pellet was re-suspended to the original volume in buffer A. Assays of activity were conducted with 100 μM NAD^+^ in buffer A containing 2 mM MgCl_2_, 2 mM CaCl_2_, 100 μM EHNA and membranes (100 to 300 μg of protein). In some cases, NAD^+^ was replaced with 100 μM ADPR. Incubations were quenched at various times by removing a 10 μL aliquot and mixing with it 100 μL of 100 mM HCl. Once reactions were stopped, they were stored at 4 °C until assay for NAD^+^. NAD^+^ standards and samples were stable in 100 mM HCl under these conditions. Once the reactions were stopped with HCl, there was no additional enzymatic activity, as confirmed by time course data. Prior to the measurement of AMP and adenosine, the pH of samples and standards was adjusted to 7.5 with a solution of 1 M Tris base.

### 4.5. Acid Extraction of Lung Tissue

The left lung was removed and quickly immersed in 1 mL of ice-cold perchloric acid (0.6 M). The tissue was immediately homogenized with 10 strokes with a homogenizer (PowerGen 700, Fisher Scientific). After removing the protein by centrifugation at 10,000 rpm for 30 min, the supernatant was adjusted to pH 7.0 with 2 M KHCO_3_. The potassium perchlorate pellet was removed by centrifugation at 10,000 rpm for 30 min and the adenosine, AMP, ADP, ATP, or NAD^+^ was measured in the supernatant.

### 4.6. ATP Assay

ATP was measured in culture media as well as in cell extracts. Media was collected (100 μL samples) at various times and quenched with 10 μL of 1 M HCl. Cells grown in 12 well plates were extracted by removing the medium and replacing it with 1 mL of 100 mM HCl. After extraction, samples were maintained at 4 °C until assay. Prior to an assay, the pH of samples was adjusted to 7.5 with 1 M Tris base. Typically, the assay for ATP measurement contained 50 μL of sample, 25 μL of lysis buffer, and 25 μL of substrate reagent. For adenosine kinase reactions containing 100 μL volumes, 50 μL of lysis buffer and 50 μL of substrate reagent were added sequentially. Luminescence was measured in a microplate reader (BMG). In general, most assays were performed in 3 steps. The first step resulted from collection of tissue or media samples and reactions were quenched with HCl. The second step involved the enzymatic conversion of adenosine or AMP to ATP and was conducted at room temperature for 3 h. Step 2 was stopped with the lysis reagent from the ATPlite kit. Step 3 was initiated by addition of the substrate reagent from the ATPlite kit.

### 4.7. NAD^+^ Assay

The NAD^+^ assay was conducted in 96 well plates and included 30 μL of sample and 120 μL of reagent. The reagent contained Na_2_HPO_4_ (100 mM), resazurin (20 μM), FMN (1 μM), 1% ethanol, alcohol dehydrogenase (30 units/mL) and diaphorase (5 μg/mL). Generation of the fluorescent product of the cycling reaction was measured at 544/590 nm excitation/emission wavelengths in a microplate reader [60].

### 4.8. CD38 Enzyme Activity Assay

CD38 enzyme activity was measured in lung homogenates, BAL cells, HASM and 16HBE cell extracts. CD38 activity was determined fluorometrically using NGD as a substrate (100 μM) and the production of cGDPR was determined at 300/405 nm excitation/emission wavelengths with a microplate reader (i.e., NGD assay) [61]. In some cases, activity was measured with a continuous assay in the microplate reader. In other cases, samples were incubated with NGD and the reactions quenched with 100 mM HCl, the pH subsequently adjusted to 7.5, and the fluorescence of the cyclic GDP-ribose was detected in the microplate reader.

### 4.9. Preparation of Adenosine Kinase

Recombinant Adenosine kinase was expressed in *E. coli* and purified as described previously [62] with some modifications. This kinase was originally cloned from the *Anopheles gambiae* mosquito and has kinetic properties including a high affinity for adenosine and acceptance of GTP as the phosphoryl donor [62]. When combined with other enzymes in a reaction mixture containing myokinase and creatine kinase, the adenosine kinase catalyzes conversion of adenosine to ATP, which is then detected with a standard luciferin/luciferase assay. The lower limit of detection is in the range of 10 to 50 nM, depending on background contaminating ATP. The adenosine kinase plasmid was transferred to the pDEST-14 vector, transformed into TOP10 competent cells, and cultured on LB-carbenicilin agar plates. The plasmid was isolated and introduced into *E. coli* BL21-AI cells for expression. The cells were grown in LB-carbenicillin at room temperature to an OD600 of 0.6 and induced with arabinose, 0.2% (*w*/*v*), for 6 h. The cells were harvested by centrifugation and stored frozen until the adenosine kinase was purified.

The bacterial cells were suspended in 10 volumes of 50 mM Na_2_HPO_4_ and disrupted by sonication, at 8 bursts of 10 s, with 30 s rest between bursts. Following sonication, cell debris was pelleted by centrifugation at 10,000 rpm for 30 min. The supernatant was collected and an equal volume of 1 M NaCl was added. Ni beads were washed in 25 mM Na_2_HPO_4_/0.5 M NaCl and added to the supernatant, 1/20th volume. The supernatant/bead mix was placed on a shaker in a cold room for 1 h. The mixture was transferred to a small plastic column to collect the beads. After the unbound extract was eluted, the column was washed with 25 mM Na_2_HPO_4_/0.5 M NaCl until protein was not detected by Bradford assay. The adenosine kinase was eluted by a step gradient of 25, 50, 100, 150, 200, 250, and 300 mM imidazole. The peak of activity usually occurred in the 100 mM imidazole fraction. Active fractions were pooled and dialyzed in 50 mM Tris, pH 7.5, 1 mM DTT. The adenosine kinase activity corresponded to fractions that were identified by Western blotting using an anti-HIS-tag antibody. The dialyzed adenosine kinase was aliquoted and stored frozen and retained activity for at least 4 months.

### 4.10. Adenosine Assay

Adenosine was measured by its sequential enzymatic conversion to ATP and detection with a luciferin/luciferase kit (Figure 1). Reactions were started by adding 50 μL of sample and 50 μL of a reagent. The basic reagent contained 50 mM Tris*HCl, pH 7.5, 10 mM MgCl_2_, 100 mM KCl, 1 mM DTT, 200 μM GTP, 10 mM creatine phosphate. ATP in a sample was measured in the absence of creatine kinase. ADP was measured from the difference in luminescence between the absence and presence of creatine kinase (50 μg/mL). AMP was measured from the difference in luminescence between the absence and presence of myokinase (rabbit muscle; 10 μg/mL) in reactions also containing creatine kinase. Adenosine was measured from the difference between the absence and presence of adenosine kinase (5 μg/mL) in reactions also containing creatine kinase and myokinase. Using this strategy adenosine, AMP, ADP, and ATP were measured in extracts from cells, lung tissue, enzyme reactions and media. In general, acid extracts from cells, reactions, and media were prepared with 100 mM HCl. Prior to assay, the pH was adjusted with 1 M Tris base. Based on preliminary data, it was determined that 3 h of incubation at room temperature was sufficient to convert the AMP and adenosine to ATP. The incubation was stopped with 50 μL of lysis reagent, and luminescence was generated after adding 50 μL of substrate reagent.

### 4.11. Uric Acid Assay

The concentration of UA was measured in BAL fluid by ELISA (Amplex Red Uric Acid; Uricase assay, Invitrogen), as per the manufacturer’s instruction. Measurements were done in BAL fluid collected from WT and CD38KO mice intranasally challenged with HDM as described above.

### 4.12. Statistical Analysis

Data were considered normally distributed by the Shapiro-Wilk normality test. One-way analysis of variance was used to determine significant differences between three or more means followed by Bonferroni post-tests. Correction for multiple comparisons were made using statistical hypothesis testing. Unpaired, two-tailed t-test was used for pairwise comparisons. Significance was set at *p* < 0.05. Data are presented as mean ± SEM. Analyses were conducted using GraphPad Prism version 8.4.3 for MAC, GraphPad Software, San Diego, CA, USA, www.graphpad.com.

## 5. Conclusions

Considering the significance of the NADases in the context of airway diseases, we conclude that a novel, non-canonical biochemical pathway is responsible for the synthesis of adenosine and UA (i.e., NAD^+^-adenosine-UA axis), and that it may be a significant contributor to the development and maintenance of airway inflammation, hyperresponsiveness, and remodeling. This pathway is important because the development of NADase inhibitors may be useful in the treatment of allergic airway diseases such as asthma. Furthermore, the therapeutic implications of defining this pathway extend to other inflammatory airway diseases. For example, high UA levels correlate with acute exacerbations of COPD, where it causes severe pro-inflammatory effects that override the antioxidant property of UA observed at lower concentrations.
